# Early Prediction of Treatment Response of Neuroendocrine Hepatic Metastases after Peptide Receptor Radionuclide Therapy with ^90^Y-DOTATOC Using Diffusion Weighted and Dynamic Contrast-Enhanced MRI

**DOI:** 10.1155/2019/1517208

**Published:** 2019-11-11

**Authors:** Thomas Weikert, Ole Christopher Maas, Tanja Haas, Markus Klarhöfer, Jens Bremerich, Flavio Forrer, Alexander Walter Sauter, Gregor Sommer

**Affiliations:** ^1^University Hospital Basel, University of Basel, Department of Radiology, Petersgraben 4, 4031 Basel, Switzerland; ^2^Cantonal Hospital St. Gallen, Clinic of Radiology and Nuclear Medicine, Rorschacher Str. 95, 9007 St. Gallen, Switzerland; ^3^Siemens Healthcare AG, Freilagerstrasse 40, 8047 Zurich, Switzerland

## Abstract

The purpose of this study was to determine if parameters derived from diffusion-weighted (DW-) and dynamic contrast-enhanced (DCE-) magnetic resonance imaging (MRI) can help to assess early response to peptide receptor radionuclide therapy (PRRT) with ^90^Y-DOTATOC in neuroendocrine hepatic metastases (NET-HM). Twenty patients (10 male; 10 female; mean age: 59.2 years) with NET-HM were prospectively enrolled in this single-center imaging study. DW-MRI and DCE-MRI studies were performed just before and 48 hours after therapy with ^90^Y-DOTATOC. Abdominal SPECT/CT was performed 24 hours after therapy. This MRI imaging and therapy session was repeated after a mean interval of 10 weeks. Up to four lesions per patient were evaluated. Response to therapy was evaluated using metastasis sizes at the first and second therapy session as standard for comparison (regressive, stable, and progressive). DW-MRI analysis included the apparent diffusion coefficient (ADC) and parameters related to intravoxel incoherent motion (IVIM), namely, diffusion (*D*), perfusion fraction (*f*) and pseudo-diffusion (*D*^*∗*^). DCE-MRI analysis comprised K_trans_, *v*_e_ and *k*_ep_. For statistical analysis of group differences, one-way analysis of variance (ANOVA) and appropriate post hoc testing was performed. A total of 51 lesions were evaluated. Seven of 51 lesions (14%) showed size progression, 18/51 (35%) regression, and 26/51 (51%) remained stable. The lesion-to-spleen uptake ratio in SPECT showed a decrease between the two treatment sessions that was significantly stronger in regressive lesions compared with stable (*p* = 0.013) and progressive lesions (*p* = 0.021). ANOVA showed significant differences in mean ADC after 48 h (*p* = 0.026), with higher ADC values for regressive lesions. Regarding IVIM, highest values for *D* at baseline were seen in regressive lesions (*p* = 0.023). In DCE-MRI, a statistically significant increase in *v*_e_ after 10 weeks (*p* = 0.046) was found in regressive lesions. No differences were observed for the transfer constants K_trans_ and *k*_ep_. Diffusion restriction quantified as ADC was able to differentiate regressive from progressive NET-HMs as early as 48 hours after PRRT. DW-MRI therefore may complement scintigraphy/SPECT for early assessment of response to PRRT. Assessment of perfusion parameters using IVIM and DCE-MRI did not show an additional benefit.

## 1. Introduction

Neuroendocrine tumors (NETs) are a heterogeneous group of mostly slowly growing malignancies originating from the cells of the diffuse neuroendocrine system and are most commonly located in the gastrointestinal tract [1]. The name is pointing at the common feature of these cells, which is the release of hormones upon neuronal input. NETs are a rare entity; however, their reported incidence has been steadily rising in the last decades, mainly due to improved diagnostic procedures [2, 3]. They represent a therapeutic challenge for three reasons: First, they stay clinically silent for a long time, which results in late diagnosis [4]. Second, for this reason, they often show hepatic metastases at the time of diagnosis and have therefore limited curative options [5, 6]. Third, many neuroendocrine tumors reluctantly respond to standard therapeutic approaches like chemotherapy and show substantial recurrence rates after surgical resection [4, 7]. Advanced therapeutic approaches are therefore warranted.

Particularly, the control of the hepatic metastases is crucial to mitigate symptoms, as the metastases can cause pain, compromise liver function, and release serotonine directly into the circulatory system, which results in carcinoid syndrome. An approach that has proven effective to control metastatic NETs is the peptide receptor radionuclide therapy (PRRT) [8–10]. It provides symptomatic relief and tumor control at the same time and is applied in more and more medical centers all over the world [11]. The mechanism of action is the selective binding of the peptide, such as DOTA(0)-D-Phe(1)-Tyr(3)-Octreotide (DOTATOC), to somatostatin receptors that are overexpressed at the surface of neuroendocrine tumor cells. The coupled *β*-emitter, e.g., Yttrium-90, finally exhibits the therapeutic effect that has to be monitored.

The standard method for assessment of therapy response in oncology is RECIST 1.1 (Response evaluation Criteria In Solid Tumors) [12, 13], which rates response based on the tumor lesions' changes in size. This approach is particularly limited in slowly growing tumors like NETs, where a robust assessment of treatment effectiveness with RECIST in accordance with the proposed frequency of tumor reevaluation in the original publication is performed after a period of several weeks after therapy [12]. This is too late for a prompt personalized adaption of therapeutic strategies and for patients with advanced and less differentiated NET grades 2 and 3. Early response assessment of those NETs within few days after PRRT can therefore be of high interest for these patients and their physicians. A currently used approach to estimate the effect of the PRRT is to measure tumor uptake and tumor-to-spleen ratio in planar posttreatment scintigraphy or SPECT/CT run a few hours to days after injection of the radiotherapeutic agent. This predominantly reflects the radiopharmaceutical's biological distribution and the density of associated receptors.

Magnetic resonance imaging (MRI) provides additional indicators to assess tissue properties in an oncologic context, mainly using diffusion-weighted MRI (DW-MRI) and dynamic contrast-enhanced MRI (DCE-MRI) [14]. DW-MRI takes advantage of the Brownian motion of water molecules to make statements about the tissue microarchitecture that is subject to changes in the course of treatment. The apparent diffusion coefficient (ADC) provides a quantitative measurement of diffusion. The concept of intravoxel incoherent motion (IVIM) further differentiates the share of DW-MRI signal that can be attributed to diffusion (high *b* values) and perfusion (low *b* values), respectively [15]. DCE-MRI on the other hand tracks the signal variation of a tissue at multiple time points after intravenous injection of a contrast medium. The widely applied Tofts model postulates two compartments (plasma space (PS) and extravascular extracellular space (EES)) and provides the quantitative parameters K^trans^ (transfer constant), *V*_e_ (fractional volume of EES), and *k*_ep_ (flux rate constant) [16]. K^trans^ (in 1 per minute) is the flow from PS to EES and physiologically represents plasma blood flow, vascular permeability, and surface area. *V*_e_ is the volume of EES (in %). *k*_ep_ is the reflux rate from EES into PS and equals *K*^trans^/*v*_e_ (in 1 per minute). The model-independent initial area under the curve (iAUC) is the integral under the enhancement curve and more robust to noise. It reflects blood flow and vascular permeability, similar to K^trans^ [17].

Both DW-MRI and DCE-MRI have proven useful for the assessment of therapy-induced changes [18, 19]. Their potential as biomarker for treatment response, before macro-anatomic changes occur, has been demonstrated in other tumors (DW-MRI: [20–26] DCE-MRI: [20, 27–29]), as well as in the special case of liver metastases of neuroendocrine tumors after transcatheter arterial chemoembolization (TACE) [30], TACE and Y-90 radioembolization [31], and selective internal radiotherapy (SIRT) [32]. However, for the increasingly applied PRRT, a multiparametric MRI evaluation is still missing.

The aim of our study was therefore to evaluate if (semi-)quantitative parameters derived from DW-MRI and DCE-MRI may be used for an early prediction of response of hepatic metastases of NETs 48 hours after PRRT with ^90^Y-DOTATOC.

## 2. Materials and Methods

### 2.1. Patients

This prospective study was performed at the Clinic of Radiology & Nuclear Medicine at the University Hospital Basel and approved by the local ethics committee (*Ethikkommission beider Basel*, case number 317/11). Informed consent was obtained from all participants. Twenty patients suffering from a neuroendocrine tumor with hepatic metastases were included. All of them presented for PRRT for the first time and were at least 18 years old. Exclusion criteria were MRI-incompatible foreign bodies (e.g., pacemakers, intracranial clips, and implants), a history of epilepsy, pregnancy, and a limited kidney function (creatinine clearance <50 ml/min).

### 2.2. Peptide Receptor Radionuclide Therapy

**Figure 1:**
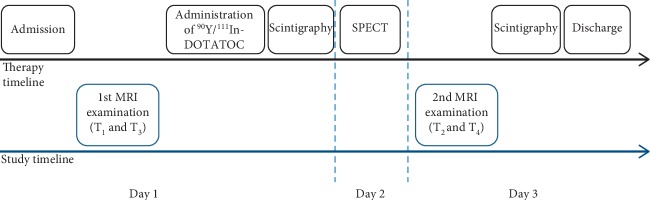
Standardized therapy and study timeline. This therapy and study cycle was repeated with a mean interval of 10 weeks (T_3_ and T_4_). In 3 of 20 patients, ^90^Y/^111^In-DOTATOC was replaced by ^177^Lu-DOTATOC in the second session because of deterioration of kidney function.

PRRT followed the standard scheme at our institution with two therapy cycles at an interval of about 10 weeks. They were supplemented by four abdominal MRI examinations for the purpose of this study performed just before (T_1_ and T_3_) and 48 hours after administration of the radiotherapeutic agent (T_2_ and T_4_). In the first session, ^90^Y/^111^In-DOTATOC was administered in all cases (100–200 mCi; mean: 168 mCi; SD: 24 mCi ≙ 3.7–7.4 GBq; mean: 6.2 GBq; SD: 0.9 GBq). ^90^Y is a *β*-emitter, ^111^In a *γ*-emitter used for SPECT imaging. In the second session, there was a switch to Lutetium (^177^Lu) in three cases (150–200 mCi; mean: 183 mCi, SD: 24 ≙ 5.6–7.4 GBq; mean: 6.8 GBq; SD: 0.9 GBq) due to deterioration of kidney function. In all other cases, ^90^Y/^111^In-DOTATOC was administered (100–200 mCi; mean: 160 mCi, SD: 31 mCi ≙ 5.6–7.4 GBq; mean: 5.9 GBq; SD: 1.1 GBq). The therapy and study timeline are shown in Figure 1.

### 2.3. SPECT/CT Imaging

SPECT/CT imaging of the abdomen was performed 24 hours after the injection of the radiotherapeutic agent. The images were acquired with a Symbia T6 SPECT/CT system (Siemens Healthineers AG, Erlangen, Germany; matrix: 128 × 128; 64 views of 20 seconds). SPECT images were reconstructed with an OSEM-based Flash 3D algorithm (8 iterations, 4 subsets, and 8 mm Gaussian filtering). For ^90^Y/^111^In-DOTATOC, a scatter and attenuation correction was performed. Additionally, at 3 and 48 hours postinjection (p.i.), scintigraphy was performed to monitor radiation exposure of the kidneys and whole body assessment, respectively.

### 2.4. Magnetic Resonance Imaging

MRI imaging was performed two times per cycle per patient on three different 1.5 Tesla scanners that were also used in the clinical routine (MAGNETOM Avanto/Symphony/Espree, all Siemens Healthineers AG, Erlangen, Germany). The baseline MRI scan (at T_1_ and T_3_) was performed shortly before the administration of the radiotherapeutic agent. A second scan (at T_2_ and T_4_) was performed 48 hours after the injection. A Gadolinium-based contrast agent (Dotarem®, Guerbet AG, 0.1 mmol Gd/kg KG) was administered intravenously. The total examination time was 30 minutes. Transversal and coronal T2-weighted images were acquired with a 2D single-shot fast spin echo sequence before administration of the contrast agent (TR/TE: 1,000/89; echo train: 256; field of view: 360 × 360 mm; matrix size: 256 × 256; number of slices: 30; slice thickness: 6 mm; spacing between slices: 7.2 mm).

#### 2.4.1. DW-MRI

2D transverse DWI images using multiple *b* values (*b* = 0, 10, 40, 70, 120, 250, 450, 700 s/mm^2^) were acquired using a prototype sequence provided by Siemens Healthineers AG in free breathing technique before contrast administration (TR/TE: 4,100/63 ms; field of view: 384 × 312 mm; matrix size: 192 × 156; number of slices: 20; slice thickness: 6 mm).

#### 2.4.2. DCE-MRI

During and after administration of the contrast agent, 17 serial transversal 3D T1-weighted datasets were acquired at multiple time points (first time frame before contrast agent injection; then every 10 seconds for the first minute p.i.; second and third minute p.i.: every 20 seconds; fourth minute until the end of acquisition at second 300 p.i.: every 30 seconds) with the following parameters: TR/TE: 2.23/0.7 ms; field of view: 360 × 315 mm; flip angle: 15°; matrix size: 128 × 112; number of slices: 30; slice thickness: 3.6 mm.

### 2.5. Image Analysis

Image analysis was performed on a dedicated workstation separate from the scanner. A maximum of four representing lesions per patient were analyzed (51 lesions in total).

The hepatic lesions were defined, and their maximum diameter was measured before and after the administration of the radiotherapeutic agent at each of the two cycles on transversal b700 DWI series using dedicated software (Mint Lesion, Mint Medical GmbH, Heidelberg, Germany). This was done by a dual board-certified radiologist and nuclear medicine physician with 11 years of professional experience (GS). The accuracy of the size measurement was determined by comparing the diameters of measurements at T_1_ and T_2_ as well as—where available—T_3_ and T_4,_ assuming no change in diameter within 48 hours. The double standard deviation then served as cutoff for a significant increase or decrease in diameter between the first (average of T_1_ and T_2_) and the second treatment cycle (average of T_3_ and T_4_, if available; otherwise: only T_3_): lesions with an increase or decrease above the cutoff value were considered progressive (PD) or regressive (RD), respectively. Lesions with size changes below the cutoff value were considered stable (SD).

#### 2.5.1. SPECT

SPECT analysis was performed on a dedicated workstation (SyngoVia, Siemens Healthineers AG, Erlangen, Germany). ROIs containing the preselected liver metastases and the spleen were defined semi-automatically based on iso-contours by a dual board-certified radiologist and nuclear medicine physician (GS). Within each ROI, the peak signal intensity (highest number of counts per 1 ccm volume within the ROI) was recorded. The lesion-to-spleen uptake ratio was then calculated for each lesion and both therapy cycles.

#### 2.5.2. DW-MRI

Diffusion analysis of the preselected liver metastases was conducted by a dual board-certified radiologist and nuclear medicine physician (GS) using SyngoVia (Siemens Healthineers AG, Erlangen, Germany) for ADC and the Medical Imaging Interaction Toolkit (MITK, German Cancer Research Center, Heidelberg, Germany) for the IVIM parameters. Apparent diffusion coefficients (ADCs) were extracted from the ADC maps that were automatically generated by the MRI scanner (mono-exponential fitting) considering the complete tumor volume. Parameters for true diffusion (*D*), pseudo-diffusion (*D*^*∗*^), and perfusion fraction (*f*) were calculated using a 3-parameter-fit according to the intravoxel incoherent motion model [33].

#### 2.5.3. DCE-MRI

Perfusion analysis was performed using the commercially available Tissue4D Package in Syngovia (Siemens Healthineers AG, Erlangen, Germany) by a 3rd year radiology resident (TW) supervised by GS. This was done in three steps: (1) Preprocessing: definition of a compartment model (Tofts model assuming a liver specific T1 relaxation time of 1000 ms); automatic motion correction; and registration with the first volume of the dynamic series as reference; definition of an arterial input function (intermediate type). (2) Calculation of perfusion parameter maps (K_trans_, *k*_ep_, *v*_e_, iAUC). (3) definition of ROIs for all previously determined lesions and extraction of perfusion parameters for each ROI (K_trans_, *k*_ep_, *v*_e_, iAUC).

### 2.6. Statistical Analysis

Statistical analysis was performed using SPSS 22 (IBM Corp., Armonk, NY). A *p*-value < 0.05 was determined to indicate statistical significance. One-way ANOVA between subjects was conducted to compare means of more than two groups. To test for homogeneity of variance, Levene's test was performed. Histograms, normal QQ-Plots, and the Shapiro–Wilk test were used to test for normal distribution of data. Post hoc comparisons were assessed with Tukey HSD test. In cases with violated assumption of homogeneity, Welch's *F*-test was used to compare means of groups and Games-Howell test was used for post hoc testing. For nonnormally distributed data, Kruskal–Wallis H test was applied to assess group differences.

## 3. Results

### 3.1. Patients

All 20 patients completed the MRI examinations at T_1_, T_2_, and T_3_. In one patient, DCE-MRI at T2 failed because of a technical error. Six patients (=30%) did not conduct the final MRI examination at T_4_. Patients' characteristics are listed in Table 1. The time distance between the first and the second treatment cycle was 10 weeks on average, with a standard deviation of ±2 weeks (range: 8–18 weeks).

### 3.2. Evaluation of Lesion Size

51 lesions were evaluated in total (see Table 2 for characteristics). When comparing the diameters measured at T_1_ vs T_2_ and (where available) T_3_ vs T_4_, a mean test-retest variability of 2.6% with a standard deviation of 3.0% was found. Based on the double standard deviation, a cutoff value of 6% was defined as the threshold for lesion size progression or regression. Lesions with size changes less than ±6% were considered stable.

When comparing lesion sizes at the first and the second treatment cycle (average of T_1_ and T_2_ vs. average of T_3_ and T_4_; in four cases, T_4_ was missing and diameter measurements at T_3_ were used), 7/51 lesions showed size progression, 18/51 regression, and 26/51 remained stable. In 13 patients (33 lesions), all lesions behaved uniformly (7x SD, 5x RD, and 1x PD). Two lesions in one of the patients with PR were too small to measure at T_3_. Mixed behavior of lesions was seen in 7 patients (4x stable + regressive lesions, as a whole rated as SD; 3x stable + progressive, as a whole rated as PD). In summary, 4 patients (20%) were rated as PD, 11 patients (55%) as SD and 5 patients (25%) as PR.

### 3.3. SPECT

**Figure 2:**
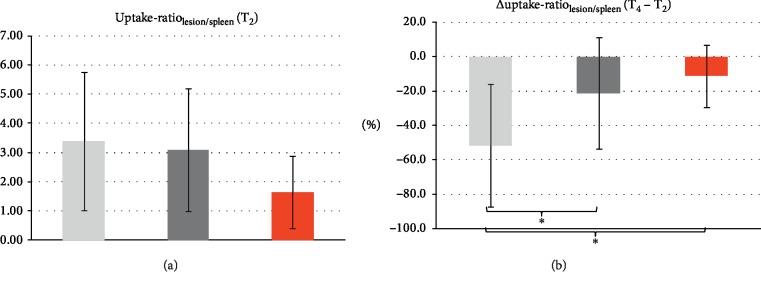
(a) Average lesion-to-spleen uptake of the radiopharmaceutical measured after the first treatment cycle in regressive (light grey), stable (dark grey) and progressive lesions (red). (b) Average difference in lesion-to-spleen uptake of the radiopharmaceutical from T_2_ to T_4_ indicating significantly higher decrease for regressive lesions (light grey) than for stable (dark grey) and progressive ones (red). The asterisks indicate statistical significance (comparison regressive vs. stable *p*=0.013; regressive vs. progressive *p*=0.021).

One patient (2 lesions) had to be excluded from the analysis of SPECT data because of splenectomy. The lesion-to-spleen uptake ratio measured at time point T_2_ was lower in progressive lesions (1.6 ± 1.2) than in stable (3.1 ± 2.1) and regressive ones (3.4 ± 2.4; Figure 2(a)). However, these differences were not statistically significant (*F* = 1.8, *p*=0.184). There were also no statistically significant group differences at T_4_ (*F* = 1.8, *p*=0.176).

When analyzing the relative differences in lesion-to-spleen uptake ratio between T_1_ and T_3_, we found a strong decrease for regressive lesions (−52% on average) and smaller decreases for stable (−21%) and progressive lesions (−11%). The difference of the means in the three groups was statistically significant (*F* = 5.8, *p*=0.006; Figure 2(b)). Post hoc comparisons using the Tukey HSD test revealed statistically significant differences when comparing the groups of regressive vs. stable lesions (*p*=0.013) and regressive vs. progressive lesions (*p*=0.021). However, no statistically significant difference was found between the groups of stable vs. progressive lesions (*p*=0.743).

### 3.4. Diffusion Weighted Imaging

**Figure 3:**
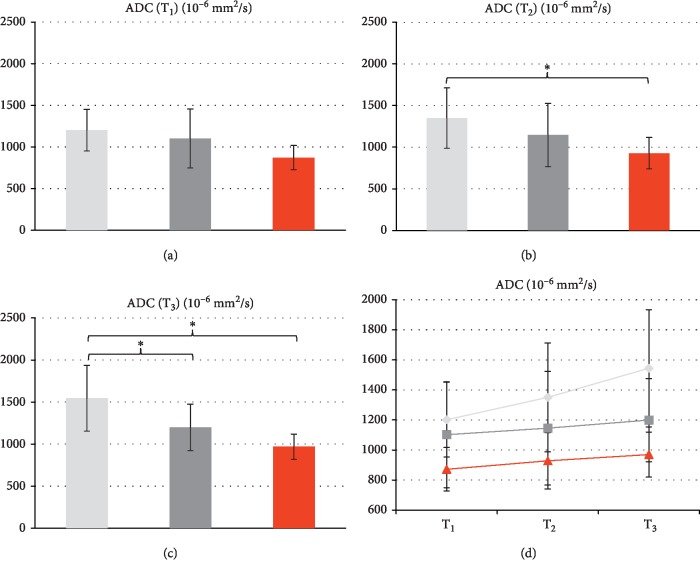
Average ADC values measured at time points T_1_ (a), T_2_ (b), and T_3_ (c) in lesions that were identified as regressive (light grey), stable (dark grey) and progressive (red) at long-term follow-up (T_3_). Statistically significant differences are seen between regressive and progressive lesions at time point T_2_ 48 hours after therapy (*p*=0.027) and between regressive lesions and the two other groups at time point T_3_ around 10 weeks after therapy (regressive vs. stable: *p*=0.003; regressive vs. progressive: *p* < 0.001). (c) Evolution of ADC values from T_1_ via T_2_ to T_3_ for the three subgroups of lesions. Evidently, the increase in ADC that is measured between T_1_ and T_3_ for all subgroups already manifest as a trend at T_2_, as early as 48 hours after treatment. It is most pronounced in regressive lesions. The asterisks indicate statistical significance.

For ADC values before treatment, ADC(T_1_) were highest in regressive lesions (1201 ± 249 × 10^−6^ mm^2^/s), followed by stable (1102 ± 354 × 10^−6^ mm^2^/s) and progressive ones (872 ± 145 × 10^−6^ mm^2^/s) (Figure 3(a)).

The group differences regarding ADC at T_1_ were not statistically significant (*F* = 3.1, *p*=0.056). However, at T_2_ (*F* = 4.0, *p*=0.026) and T_3_ (*F* = 10.4, *p* < 0.001), ANOVA showed statistically significant differences in mean ADC. Post hoc comparisons with Tukey HSD revealed, that at T_2_, only the groups with regressive vs. progressive lesions showed statistically significant differences in ADC (*p*=0.027), whereas at T_3_, differences of the groups with regressive vs. stable (*p*=0.003) and regressive vs. progressive lesions (*p* < 0.001) were statistically significant.

When comparing the differences in ADC before treatment and after 10 weeks ADC(T_3_)-ADC(T_1_), a higher increase in ADC was seen in regressive lesions (346 ± 322 × 10^−6^ mm^2^/s) than in stable (97 ± 192 × 10^−6^ mm^2^/s) and progressive lesions (98 ± 38 × 10^−6^ mm^2^/s). This difference was statistically significant (*F* = 6.2, *p*=0.004). Post hoc testing showed that only the comparison of regressive vs. stable lesions was statistically significant (*p*=0.040), while the comparison of regressive vs. progressive lesions showed a statistical trend (*p*=0.058).

**Figure 4:**
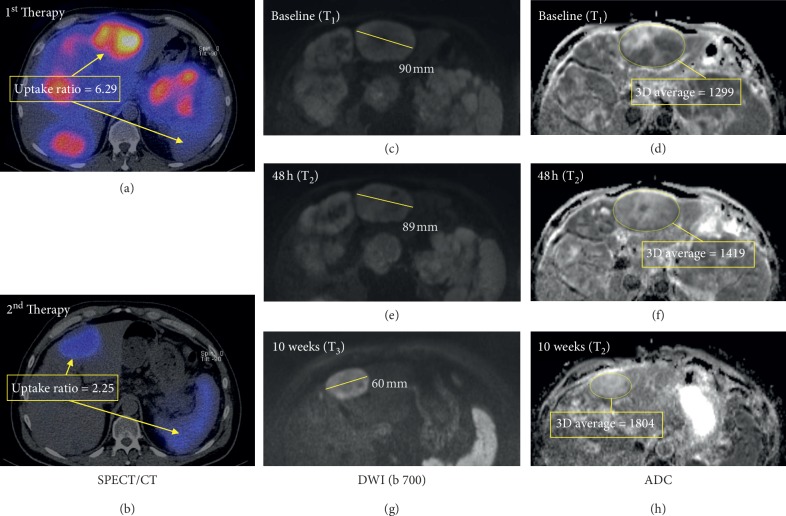
Cross-sectional imaging studies of a 53 y/o male with NET of pancreatic origin with hepatic metastases treated with two cyles of PRRT (195 mCi and 170 mCi of ^90^Y/^111^In -DOTATOC). The patient responded well to the first therapy with SPECT/CT images (a, b) showing a markedly decreasing uptake ratio after the 2^nd^ therapy as compared with the 1^st^ treatment cycle. Correspondingly, the size of the lesions as documented by the *b* = 700 s/mm^2^ diffusion-weighted MR images (c, e, g) showed an important decrease in size 10 weeks after treatment. In the ADC maps (d, f, h), an increase is seen already at T_2_, 48 h after initiation of therapy as an early sign of treatment response.

Figure 3(d) displays the evolution of the average ADC values from time point T_1_, via T_2_ to T_3_. Evidently, the abovementioned increase in ADC that is measured between T_1_ and T_3_ already manifest as a trend at T_2_, as early as 48 hours after treatment. The differences T_2_ − T_1_ between the three subgroups, however, were not yet statistically significant (*F* = 1.053; *p*=0.357). A sample-case illustrating the abovementioned behavior of ADC as an early indicator of treatment response in a patient with NET-HM of pancreatic origin is shown in Figure 4.

**Figure 5:**
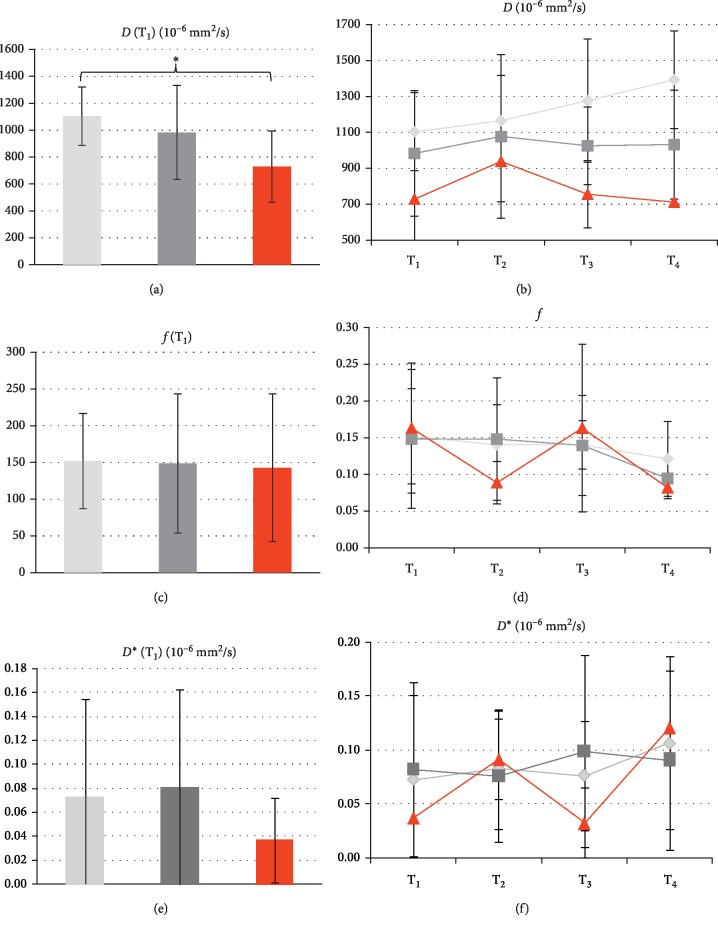
IVIM evaluation results for *D* (a, b), *f* (c, d), and *D*^*∗*^ (e, f) and the three classes of lesions (light grey: regressive; dark grey: stable; red: progressive). The bar plots in (a, c, e) show the values obtained before the initiation of the treatment (T_1_), while graphs in (b, d, f) show the evaluation of the values over time (from T_1_ to T_4_). Note the analogous behavior of *D* as compared with the ADC values displayed in Figure 3. A statistically significant difference is seen only for *D* when comparing regressive vs. progressive lesions at T_1_ (asterisk; *p*=0.017).

The values obtained for the diffusion coefficient *D* using the IVIM method corresponded well to the ADC values, yet they were associated with larger measurement errors (Figures 5(a) and 5(b)). At T_1_, *D* was highest for regressive lesions (1103 ± 217 × 10^−6^ mm^2^/s), followed by stable (983 ± 350 × 10^−6^ mm^2^/s) and progressive ones (728 ± 264 × 10^−6^ mm^2^/s). The group differences were statistically significant (*F* = 4.1, *p*=0.023). A more detailed look with post hoc testing showed that only the comparison of regressive vs. progressive lesions (*p*=0.017) was statistically significant. The evolution of *D* values over time from T_1_ to T_4_ was also comparable to that of ADC with a continuous increase seen for regressive lesions. However, when comparing the difference in *D* at T_3_ minus T_1_ and at T_2_ minus T_1_, no statistically significant difference between the groups was found (*F* = 1.7, *p*=0.198 and *F* = 1.1, *p*=0.341, respectively).

The perfusion fraction *f* (Figures 5(c) and 5(d)) did not show any significant differences between the three groups of lesions at T_1_ (*F* = 0.1, *p*=0.912), T_2_ (*F* = 1.8, *p*=0.185), and T_3_ (*F* = 0.9, *p*=0.424). Measured values ranged from 0.08 in progressive lesions at T_4_ to 0.16 in progressive lesions at T_1_ and T_3_. Interestingly, a drop of perfusion fraction was observed for the progressive lesions at T_2_ and T_4_; however, the differences T_2_ − T_1_ (*F* = 0.1, *p*=0.914) and T_3_ − T_1_ (*F* = 0.03, *p*=0.968) were not statistically significant.

The pseudo-diffusion coefficient *D*^*∗*^ (Figures 5(e) and 5(f)) did not show any significant variation between the three subgroups at any time point as assessed by the Kruskal–Wallis H test due to nonnormally distributed data (T_1_: *χ*^2^ = 1.6, *p*=0.4; T_2_: *χ*^2^ = 0.3, *p*=0.847; T_3_: *χ*^2^ = 4.9, *p*=0.088). Visually, the pattern of variation of *D*^*∗*^ over time was opposite to that of *f*, showing an increase in *D*^*∗*^ for progressive lesions at T_2_ and T_4_. Yet these effects were not statistically significant (difference T_3_ − T_1_: *p*=0.872; T_2_ − T_1_: *p*=0.556). Measured values for *D*^*∗*^ ranged from 320 × 10^−6^ mm^2^/s for progressive lesions at T_3_ to 1208 × 10^−6^ mm^2^/s for progressive lesions at T_4_. In general, the measurement values for *f* and *D*^*∗*^ were subject to a rather large variability in between subjects and lesions (see error bars in Figure 5).

### 3.5. Perfusion Weighted Imaging

**Figure 6:**
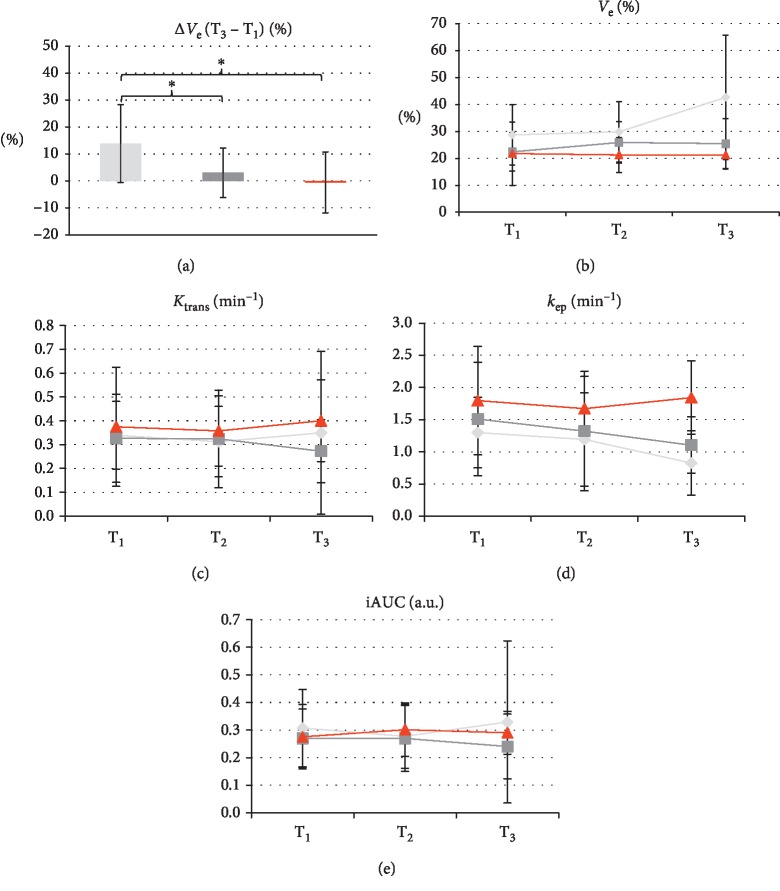
DCE-MRI evaluation results for *v*_e_ (a, b), K_trans_ (c), *k*_ep_ (d), and iAUC (e) and the three classes of lesions (light grey: regressive; dark grey: stable; red: progressive). The plots in (a) shows the variation of *v*_e_ over 10 weeks after therapy (from T_1_ to T_3_) with significant differences for regressive vs stable (*p*=0.049) and regressive vs progressive lesions (*p*=0.036; asterisks). The graphs in (b–e) demonstrate the variation of the four parameters over time, indicating a significant increase of *v*_e_ in regressive lesions as compared with the two other groups at T_3_ (b), but no significant differences in behavior between the three classes of lesions for K_trans_ (c), *k*_ep_ (d), and iAUC (e).

Because of the high sensitivity of DCE-MRI to breathing artifacts, five patients (15 lesions) had to be excluded from this part of the analysis. The values obtained for extracellular volume fraction (*v*_e_) are displayed in Figures 6(a) and 6(b). Statistically significant group differences in *v*_e_ were seen at T_3_ (*F* = 3.8, *p*=0.046) and for the difference T_3_ − T_1_ (*F* = 4.1, *p*=0.025), i.e., between the first and second treatment cycles. Post hoc analyses revealed differences between *v*_e_ at T_3_ for regressive vs. progressive (*p*=0.011) and regressive vs. stable lesions (*p*=0.010) as well as for regressive vs. progressive (*p*=0.036) and regressive vs. stable lesions (*p*=0.049) regarding the difference T_3_ − T_1_. This corresponds well to the increase in ADC and *D* seen in Figures 3(d) and 5(b), respectively. At time points T_1_ and T_2_, *v*_e_ was also higher for regressive lesions than for stable and regressive ones, but these differences were not statistically significant (*p*=0.209 and *p*=0.146, respectively). No statistically significant differences were seen when comparing T_1_ and T_2_ (*p*=0.335).

The measured values for the transfer constants K_trans_ and *k*_ep_ are displayed in Figures 6(c) and 6(d). The parameter K_trans_ showed no statistically significant group differences at any point in time (T_1_: *p*=0.835; T_2_: *p*=0.868; T_3_: *p*=0.327) and for the differences T_3_ − T_1_ (*p*=0.611) and T_2_ − T_1_ (*p*=0.921). The parameter *k*_ep,_ which equals *K*_trans_/*v*_e_, showed no statistically significant group differences at time points T_1_ (*p*=0.477) and T_2_ (*p*=0.495), as well as for the differences T_3_ − T_1_ (*p*=0.271) and T_2_ − T_1_ (*p*=0.927). However, at T_3_, there was a statistically significant difference between the response groups (*F* = 9.4, *p*=0.001) that was driven by the differences of regressive vs. progressive (*p*=0.001) and stable vs. progressive lesions (*p*=0.04), as revealed by post hoc analysis. For iAUC, there was no statistically significant variation at any point in time (T_1_: *p*=0.687; T_2_: *p*=0.816; T_3_: *p*=0.413) and neither for the differences T_3_ − T_1_ (*p*=0.539) and T_2_ − T_1_ (*p*=0.600).

## 4. Discussion

The aim of this study was to investigate if diffusion-weighted and contrast-enhanced magnetic resonance imaging can help to assess early tumor response to peptide receptor radionuclide therapy (PRRT) with ^90^Y-DOTATOC in neuroendocrine hepatic metastasis (NET-HM). For this purpose, MRI was performed immediately before initiation of PRRT, as well as 48 h and 10 weeks after treatment. Results were compared with posttherapy ^90^Y/^111^In-DOTATOC SPECT.

The most notable result of our study is that ADC can differentiate regressive from progressive lesions as early as 48 h after therapy. Lesions that were later found to be regressive in size had a significantly higher ADC at this time point than progressive lesions. The fact that apoptosis-inducing therapies like PRRT lead to an increase in ADC values due to the swelling of cells, tumor lysis, and necrosis is well known: Wulfert et al. found an increase in both responding and nonresponding lesions in their study on 38 hepatic NET after intra-arterial treatment with ^90^Y/^111^In -/^177^Lu-DOTATOC [34], which is well in line with the increase in ADC that is observed after 10 weeks in our study for all groups of lesions. However, an effect on ADC that allows for predicting treatment response of PRRT in NET-HM as early as 48 h after therapy has not been reported yet. The ADC did show a continuous increase from regressive to progressive metastases (RD > SD > PD). The differences between RD/SD and SD/PD were not statistically significant, most likely due to the relatively small number of metastases analyzed.

As an extension to previously published studies on DWI in NET-HM, our study also included an analysis of perfusion effects in DW-MRI using the concept of IVIM. In this analysis, no additional effects to standard DWI were seen, except for the fact that the average *D* observed in regressive lesions before therapy was significantly higher than in progressive lesions. This effect, which is also seen as a trend for ADC in standard DW-MRI, is also in accordance with the results of Wulfert et al., who described a significant correlation between baseline ADC and decrease in lesion size after therapy [34]. Interestingly, a drop of perfusion fraction accompanied by an increase in *D*^*∗*^ was observed for the progressive lesions at T_2_ and T_4_. However, this was not statistically significant and may be due to the considerable measurement variability present in our IVIM analysis.

For DCE-MRI, the only significant observation was an increase in the extracellular volume fraction *v*_e_ that occurred in regressive lesions 10 weeks after therapy, while for stable or progressive lesions, *v*_e_ did not change statistically significantly. This behavior of *v*_e_ probably reflects the well-known effects of the treatment in terms of tumor lysis, necrosis, and fibrosis. It is closely related to the changes in ADC described above and is completely in line with previous results of Atuegwu and colleagues [35]. Regarding K^trans^ and *k*_ep_, no significant effects of the treatment on these parameters was observed. This might be due to the low number of patients. Another reason might be the variability in quantitative DCE-MRI [36]. Our findings are in line with the quite heterogeneous results published on this topic. Zahra and colleagues reported that higher baseline K^trans^ and *k*_ep_ were positively correlated with tumor response of cervix cancer to radiation [37]. Higher baseline K^trans^ and *k*_ep_ in the responder group were also reported by Tao et al. who assessed the response 36 patients with NSCLC to chemo-radiotherapy [38]. Gu et al. did not find baseline DCE-MRI parameters useful for the discrimination of responders vs. nonresponders; however, this study as many others operated on small sample sizes (*n* = 8; [39]).

Finally, the response of the NET-HM to PRRT was also assed with ^90^Y-/^111^In-DOTATOC SPECT, which displays the density of the somatostatin-receptor subtype 2 (SSR2) in tissue and is used as an indicator of expected treatment response of PRRT. As expected keeping in mind previous literature [40], a drop in lesion-to-spleen uptake ratio between the first and the second treatment cycles was seen that was significantly higher for regressive lesions than for stable or progressive ones. The uptake ratio after treatment cycle one, however, was not predictive of therapy response in our small group of patients, which is most likely a result of low statistical power.

Our results are affected by some limitations: First, the small sample size that is due to the exploratory nature of or study, which aimed at identifying parameters of potential prognostic value rather than carving out the exact prognostic value of each parameter. Second, the choice of the reference standard in terms of morphologic response criteria, which were calculated from the test-retest variation of size measurements over 48 h. We are well aware that the threshold of ±6% which resulted from our calculations is far below the thresholds for response assessment proposed in RECIST (12), mRECIST [41], or the EASL response criteria [42]. It was chosen to be more sensitive for changes in diameter, as it is well known that the usually applied response criteria for solid tumors are limited in slowly growing tumors like NET [40]. As the rates of treatment response in our study cohort (20% PD, 55% SD, 25% RD) are consistent with previous reports on PRRT [43], we are convinced that the ±6% threshold eventually represents a reasonable value in this setting. Third, no long-term follow-up imaging and patient survival data were available as reference standard due to the fact that patients underwent follow-up imaging mostly at other centers. Fourth, some series had to be excluded from DW-MRI and DCE-MRI analyses because of imaging artifacts. These may be avoided by the use of modern sequences that apply respiratory triggering or self-gating technologies. Fifth, one of the assumptions of Tukey HSD test is independence of observations. However, one can argue that the observations are not fully independent due to the fact that in some cases, up to four lesions were measured in one liver. Due to the fact that NET is a rare entity, we did not want to exclude data by including only one metastasis per liver. Therefore, we chose to accept this statistical limitation. Finally, no double reading was performed, so interrater variability is not accounted for in our analysis.

## 5. Conclusion

In conclusion, diffusion restriction quantified as ADC was the most sensitive MRI parameter to predict treatment response of NET after PRRT among those investigated in our study. ADC was able to differentiate regressive from progressive NET-HMs as early as 48 hours after PRRT. DW-MRI therefore may complement scintigraphy/SPECT for early prediction of treatment response in the framework of PRRT. Assessment of perfusion parameters using IVIM and DCE-MRI did not show an additional benefit in our study but may nevertheless be useful to investigate pathophysiological aspects of PRRT in a preclinical setting.

## Figures and Tables

**Table 1:** Patient characteristics.

Characteristic	Patients (*n*=20)
Age, years	
Mean (SD)	59.2 (9.9)
Median	61
Gender, number of patients	
Male	10
Female	10
Primary tumor site, number of patients	
Small bowel	10
Pancreas	8
Lung	1
Unknown	1
Histology, number of patients	
G1/G2	14
G3	2
Unknown	4
Treatment, number of patients	
Only ^90^Y-DOTATOC	17
Switch to ^177^Lu-DOTATOC in second therapy session	3

**Table 2:** Lesion characteristics. Baseline size calculated as (T_1_ + T_2_)/2, size after therapy as (T_3_ + T_4_)/2. In four cases, the T_4_ measurement was not available and therefore the diameter measured at T_3_ used.

Characteristic	Patients (*n*=20)
Lesions per patient	
1	3
2	7
3	6
4	4
Size of the lesions, longest diameter	
Baseline, mean (SD)	41 mm (27 mm)
After therapy, mean (SD)	36 mm (24 mm)
Lesion response	
Regressive (number/mean diameter change)	18/−26.0%
Stable (number/mean diameter change)	26/−0.6%
Progressive (number/mean diameter change)	7/+10.3%

## Data Availability

A large part of the data are patient data and thus confidential. Upon request, a minimal anonymized dataset will be available to interested researchers.
